# Psychological Impact of the COVID-19 Pandemic on Licensed Full-Time Practicing Nurses Undertaking Part-Time Studies in Higher Education: A Cross-Sectional Study

**DOI:** 10.3390/ijerph18168569

**Published:** 2021-08-13

**Authors:** Siu-Ling Chan, Naomi Takemura, Pui-Hing Chau, Chia-Chin Lin, Man-Ping Wang

**Affiliations:** School of Nursing, The University of Hong Kong, Hong Kong 999077, China; naomitak@connect.hku.hk (N.T.); phchau@graduate.hku.hk (P.-H.C.); mpwang@hku.hk (M.-P.W.)

**Keywords:** COVID-19 pandemic, licensed full-time practicing nurses, part-time studies in higher education, GAD-7, PHQ-2, CD-RISC-10, stress-related questions, brief-COPE, resilience, mindfulness

## Abstract

Frontline nurses face an unpreceded situation with the coronavirus disease (COVID-19) pandemic, and many report suffering from physical and psychological stress. This online, cross-sectional survey used questionnaires, such as the Generalized Anxiety Disorder (GAD-7) questionnaire, the Patient Health Questionnaire (PHQ-2), the Connor–Davidson Resilience Scale, stress-related questions, and Brief Coping Orientation to Problems Experienced (Brief-COPE), to determine the psychological impact of COVID-19 on licensed full-time practicing nurses undertaking part-time studies in higher education. Recruitment commenced from August to September 2020; 385 students were approached, and 124 completed the survey (response rate: 32%). Most of the respondents were frontline nurses working in public sectors (89.5%), 29% of whom reported symptoms of depression, and 61.3% reported mild to severe levels of anxiety. The GAD-7 was significantly associated with the resilience score (β = −0.188; *p* = 0.008) and exhaustion (β = 0.612; *p* < 0.001). The PHQ-2 was significantly associated with ‘anxiety about infection’ (β = 0.071; *p* = 0.048). A lower anxiety level was significantly associated with a higher resilience level and a lower level of exhaustion, and a lower depression level was significantly associated with a lower anxiety about infection. Nursing programs incorporating resilience building may mitigate psychological distress of the study population.

## 1. Introduction

A rapid large-scale outbreak of coronavirus disease 2019 (COVID-19), caused by the 2019 novel coronavirus (SARS-CoV-2), first occurred in December 2019 and has continued to surge exponentially worldwide. Following more than 142 million confirmed cases and more than 3 million deaths globally, the pandemic has plunged the public into isolation, depression, and anxiety due to its characteristically high person-to-person transmission [1]. In response to the COVID-19 pandemic, the Hong Kong Government has adopted a variety of stringent public health measures that included border restrictions, compulsory quarantine of certain persons arriving at Hong Kong, temporary closure of non-essential businesses, work from home arrangements, suspensions of schools/universities, and social distancing, in order to mitigate COVID-19 transmission. Though a total lockdown was not inflicted, most of the leisure venues were closed inevitably [2]. With such unforeseen changes to daily living, they have adversely affected the mental health of Hong Kong people. In a general population survey, it has been reported that among 500 respondents in Hong Kong, 19% had depression, 14% had anxiety, and 25.4% had deterioration in mental health since the pandemic [3].

COVID-19 follows other significant public health emergencies, including the Severe Acute Respiratory Syndrome, Ebola, and Middle East Respiratory Syndrome epidemics, and challenges the mental health of healthcare workers [4]. During the COVID-19 pandemic, many countries have locked down due to the high prevalence of confirmed cases and the impact on the physical and mental health of healthcare workers [5]. Another survey reported that there was widespread disruption of many kinds of critical mental health services across six regions of World Health Organization [6]. Worldwide, frontline workers face an unpreceded situation and suffer from immense physical and psychological stress [7]. Exposure to excessive stress for prolonged periods has long-term harmful consequences on the emotional and mental well-being of frontline workers [7]. In a large cross-sectional survey-based study on frontline healthcare workers in the U.S., 39% of them experienced COVID-19 related posttraumatic stress disorder, major depressive disorder, and generalized anxiety disorder [8].

The COVID-19 pandemic has caused unprecedented challenges to health professionals, particularly among nurses. A systematic review revealed that the psychological impact was greater among frontline nurses than in the rest of the health professionals [9]. In a nationwide survey of an Asian country, the psychological distress of frontline nurses was generally serious [10]. In Hong Kong, many practicing nurses at the frontline in the public sector are working in case or surveillance wards to take care of suspected or confirmed COVID-19 cases [11]. They are constantly concerned about bringing the virus to their own homes, the shortage of protective equipment, and feelings of incapability when faced with critically ill patients [12].

Given the pressure in healthcare settings, it is anticipated that the stress level faced by those licensed full-time practicing nurses undertaking part-time studies in higher education is even higher. Vigilant preventive measures have been adopted by tertiary institutions, such as changing all face-to-face teaching and learning activities and assessments to online mode. However, this may hinder their learning progress, as they may need time to adapt to new teaching and learning platforms [13]. With this disruption, they were considered to be at particular risk of suffering from stress resulting from both their full-time work and part-time studies. In Hong Kong, several nursing institutions have been offering the top-up degree, as well as master degree, for registered nurses for years in order to enhance their skills in providing quality nursing care in evidence-based practice. They were the post-registration undergraduates and postgraduates. This was not unique to Hong Kong but also present in some other well-developed countries, such as Australia, New Zealand, USA and Canada, and the UK [14]. It is vital for practicing nurses to maintain good mental health to better control infectious disease [4]. By understanding how this distress could be propagated during the COVID-19 pandemic, particularly among the post-registration undergraduates and postgraduates, who were licensed full-time practicing nurses undertaking part-time studies, it was hoped that contributing factors could be identified, and, thus, possible interventions could be proposed to address their psychosocial well-being. Interventions, such as building resilience and positive coping, are well known for their effectiveness in enhancing psychosocial health [15,16,17]. Resilience is defined as the ability to return to a state of normalcy from adversity and hold a positive outlook towards the future [16]. Resilience can be viewed as a measure of stress-coping ability in response to hard time and is used as a target for treating depression, anxiety and stress problems [15]. Coping is defined broadly as an effort to minimize the distress associated with negative life events [17]. The styles of coping are described in terms of approach coping and avoidant coping. Approach coping is associated with more helpful responses to adverse situation, including adaptive practical adjustments, better physical health outcomes, and more stable emotional responses. Alternatively, avoidant coping has been shown to be less effective in managing anxiety; it involves attempts to avoid stressful condition via social distraction from the situation rather than actively dealing with it [17].

This study identified the psychological impact of COVID-19 on licensed full-time practicing nurses undertaking part-time studies in higher education. The objective was to examine the associations between psychological distress and stress-related factors, resilience, coping, and sociodemographic factors.

## 2. Materials and Methods

### 2.1. Participants and Recruitment

This was an online cross-sectional survey. The researchers in this study were the teaching faculty in the author’s institution and they taught the licensed full-time practicing nurses studying part-time undergraduate or postgraduate programs. These nursing students were recruited using convenience sampling by sending a Qualtrics survey link via their email accounts from 3 August 2020 to 15 September 2020. The permissions from the Directors of part-time undergraduate and postgraduate programs were sought before the study commenced. The participants provided online-informed consent before filling in the questionnaire by clicking ‘yes’ to indicate their willingness to participate. Voluntary participation and data confidentiality were ensured.

Ethics approval was obtained from the Institutional Review Board of the University of Hong Kong/Hospital Authority Hong Kong West Cluster (UW20-500). The study was conducted in accordance with the Declaration of Helsinki.

### 2.2. Instruments

Given that all respondents were university students and were used to English as a medium of instruction in the university learning environment, all instruments were prepared in English.

#### 2.2.1. Generalized Anxiety Disorder (GAD-7)

The 7-item Generalized Anxiety Disorder Scale was designed to identify probable cases of GAD and to assess symptom severity [18]. It describes the most prominent diagnostic features of the Diagnostic and Statistical Manual of Mental Disorders-IV diagnostic criteria A, B, and C for GAD [19]. The respondents were asked how often, during the last 2 weeks, they had been bothered by each of the seven core symptoms of GAD. The responses were rated as ‘not at all’, ‘several days’, ‘more than half of the days’, and ‘nearly every day’, and were scored from 0 to 3. The total score ranges from 0–21, with scores of ≥5, ≥10, and ≥15 denoting mild, moderate, and severe levels of anxiety, respectively. The GAD-7 attained high reliability and validity [18].

#### 2.2.2. Patient Health Questionnaire (PHQ-2)

The PHQ-2 is the first two items of the PHQ-9, which is the full depression scale of the PHQ [20]. The core question was: ‘Over the last 2 weeks, how often have you been bothered by any of the following problems’? The two items are: ‘little interest or pleasure in doing things’ and ‘feeling down, depressed, or hopeless’. The responses were rated as ‘not at all’, ‘several days’, ‘more than half of the days’, and ‘nearly every day’, and were scored from 0 to 3. The total score ranges from 0–6, with a score of ≥3 indicating a positive screen for depressive symptoms.

#### 2.2.3. Resilience

The Connor–Davidson Resilience Scale (CD-RISC-10) is a self-administered questionnaire for measuring resilience, i.e., the ability to bounce back from the variety of challenges arising in life during the past month [21]. It consists of 10 items, each of which is scored on a 5-point Likert scale from 0, indicating not true at all, to 4, indicating true nearly all time. The total score of the questionnaire is the sum of the score for each item (range, 0–40), with higher scores reflecting a higher level of resilience. The CD-RISC-10 showed high internal consistency (Cronbach α = 0.88) [22].

#### 2.2.4. Stress-Related Questions

The stress-related questions consisted of 19 items examining how often respondents experienced the 19 items during the pandemic. Each item is rated on a 4-point Likert scale (0, never; 1, rarely; 2, sometimes; 3, always). These questions have been used in studies on severe acute respiratory syndrome (SARS) [23] and H1N1 [24]. In previous studies, the 19 items were categorized into four factors (anxiety about infection, exhaustion, workload and feeling protected), which demonstrated high internal consistency with Cronbach α ranges of 0.69–0.83 [24]. Based on this validated instrument, two more questions were added to assess if there were any psychological impact of part-time studies among the participants during the pandemic: ‘burden of change to online teaching and learning mode’ and ‘burden of change in course assessment’. After adding these two questions, it demonstrated a high internal consistency with Cronbach α as 0.87. The scores for each factor were interpreted separately. Higher scores in factors for anxiety about infection, exhaustion, workload, and part-time study indicated a higher level of stress, while a higher score in the factor for feeling protected indicated a lower level of stress [24].

#### 2.2.5. Coping Behavior

The Brief Coping Orientation to Problems Experienced (Brief-COPE) is a 28-item questionnaire assessing trait coping (the usual way in which people cope with stress in everyday life) and state coping (the way in which people cope with a specific stressful situation) [25]. The Brief-COPE comprises 14 dimensions, with two items per dimension, measuring self-distraction, active coping, denial, substance use, use of emotional support, use of instrumental support, behavioral disengagement, venting, positive reframing, planning, humor, acceptance, religion, and self-blame. Each question is rated on a 4-point Likert scale, where 1 denotes ‘I have not been doing this at all’, and 4 denotes ‘I have been doing this a lot’; higher scores indicate greater adoption of the coping strategies. Each dimension of the Brief-COPE demonstrated acceptable internal consistency, ranging from 0.50–0.90 [25]. Two major components underlying the scale were further identified [17]. The first component was represented by avoidance coping, with 6 items, including denial, substance use, venting, behavioral disengagement, self-distraction, and self-blame [17]. The other component was represented by approach coping, with 6 items, including active coping, positive reframing, planning, acceptance, seeking emotional support, and seeking informational support [17]. The mean scores of the approach and avoidance coping items were used in the analysis, with higher scores indicating a stronger tendency to adopt the coping behaviors. The internal consistency of this instrument was acceptable for both approach coping (Cronbach’s a = 0.71) and avoidant coping (Cronbach’s a = 0.70) [26].

#### 2.2.6. Sociodemographic and Other Characteristics

Personal characteristics, including age, sex, year of study, household environment (e.g., number of people living within the household), and online learning pattern (e.g., number of hours spent on online learning each week in the last semester) were assessed. Information about the work environment (e.g., ever been deployed to a COVID-19 case ward/surveillance ward) and work experience during COVID-19 (e.g., average days in contact with suspected/confirmed COVID-19 cases) was also collected.

### 2.3. Statistical Analysis

Descriptive statistics were used to analyze the sociodemographic characteristics and outcome measures of the respondents. Linear regression models were adopted to examine the differences in psychological distress (anxiety and depression) between sociodemographic and other related variables. Adjusted models were also performed by controlling for demographic factors, such as age, sex, marital status, working experience, and program of study. Statistical analysis was performed using SPSS statistical software, version 26.0 (IBM Corporation: Armonk, NY, USA). All statistical tests were two-tailed with a 5% level of statistical significance.

## 3. Results

### 3.1. Sociodemographic and Other Characteristics of Respondents

A total of 385 students were approached, among whom 124 completed the online survey, with a response rate of 32%. Table 1 shows the respondents’ sociodemographic and other characteristics. The mean age of the respondents was 29.5 (SD = 5.4) years. Less than half of the respondents were studying a Master’s program (48.4%). Most respondents were female (87.1%), single (78.2%), and working in public hospitals or the government Department of Health (89.5%). Over half of the respondents had work experience of <1 year, 5 years after obtaining their RN license (58.9%). Nearly half of the respondents (46%) had spent at least 14 days caring for patients with suspected or confirmed COVID-19, and over half of the respondents had spent <9 h on online learning every week (53.2%). Most respondents (86.3%) had not attended mindfulness or resilience building workshops, while over one-third (38.7%) of them were interested in attending the workshop.

### 3.2. Respondents’ Mental Health and Other Outcomes

Table 2 shows the mental health and other outcomes of the respondents. The mean (SD) GAD-7, PHQ-2, and resilience scores were 6.14 (4.02), 1.8 (1.28), and 23.53 (5.59), respectively. The mean score of stress-related factors in terms of anxiety about infection, exhaustion, workload, feeling of being protected, and part-time study were 12.27 (5.06), 5.92 (2.95), 3.59 (1.57), 2.64 (1.56), and 3.03 (1.16) respectively. The mean score of avoidance coping and approach coping were 23.33 (4.95) and 31.14 (5.39), respectively.

### 3.3. Factors Associated with GAD-7

Table 3 displays the unadjusted and adjusted regression models for the GAD-7. In the unadjusted models, resilience score (β = −0.371; 95% confidence interval (CI) = −0.481, −0.260; *p* < 0.001), avoidance-coping score (β = 0.269; 95% CI = 0.132, 0.406; *p* < 0.001), and stress-related factors in terms of anxiety about infection (β = 0.427; 95% CI = 0.307, 0.547; *p* < 0.001), exhaustion (β = 0.875; 95% CI = 0.687, 1.062; *p* < 0.001), workload (β = 1.225; 95% CI = 0.821, 1.629; *p* < 0.001), and part-time study (β = 1.438; 95% CI = 0.873, 2.002; *p* < 0.001) were significantly associated with the GAD-7. In the adjusted model, only resilience score (β = −0.188; 95% CI = −0.327, −0.050; *p* = 0.008) and one stress-related factor in terms of exhaustion (β = 0.612; 95% CI = 0.298, 0.925; *p* < 0.001) remained significant. The variance inflation factor (VIF) was < 3 for all factors, indicating the absence of multicollinearity.

### 3.4. Factors Associated with the PHQ-2

Table 4 displays the unadjusted and adjusted regression models for the GAD-7. In the unadjusted model, resilience score (β = −0.099; 95% CI = −0.135, −0.062; *p* < 0.001), avoidance-coping score (β = 0.048; 95% CI = 0.003, 0.094; *p* = 0.036), and stress-related factors in terms of anxiety about infection (β = 0.122; 95% CI = 0.083, 0.162; *p* < 0.001), exhaustion (β = 0.210; 95% CI = 0.142, 0.278; *p* < 0.001), workload (β = 0.347; 95% CI = (0.215, 0.479; *p* < 0.001), feeling protected (β = −0.191; 95% CI = −0.334, −0.048; *p* = 0.009), and part-time study (β = 0.342; 95% CI = 0.156, 0.529; *p* < 0.001) were significantly associated with the PHQ-2. In the adjusted model, only one stress-related factor in terms of anxiety about infection (β = 0.071; 95% CI = 0.001, 0.141; *p* = 0.048) remained significant. The VIF was <3 for all factors, indicating the absence of multicollinearity.

## 4. Discussion

To the best of our knowledge, this is the first study to investigate the effects of changes in the working environment and study modality on the mental health status (anxiety, depressive symptoms, stress-related factors, and resilience) and coping mechanisms of full-time nurses engaging in part-time study.

Our survey revealed that most of the respondents were frontline nurses working in public sectors (89.5%), with nearly one-third reporting symptoms of depression (29%) and more than half reporting mild to severe levels of anxiety (61.3%) during the COVID-19 pandemic. These findings were comparable with those of previous studies in mainland China [27] and the U.S. [28]. Meanwhile, the percentages of depression and anxiety among the general population in Hong Kong [3] were lower than those of the study cohort in the current study as expected. These findings were also consistent with those reported in a previous study in mainland China [29]. This was particularly true when frontline nurses carried critical roles and responsibilities in fighting COVID-19 in the healthcare settings. In facing these public health emergencies, frontline healthcare workers during the COVID-19 pandemic were at risk of developing psychological distress and other mental health symptoms. The sources of mental health burden are considered to be related to the risk of infection, uncertainty, home quarantine and social isolation, high work demand and low work control, physical exhaustion, sleep disruption, and weakened immune system due to high levels of stress [30]. In addition to part-time study, the ever-increasing number of suspected and confirmed cases have further contributed to the mental burden of this study cohort.

Our findings showed that the resilience score was significantly negatively associated with the level of anxiety, which implies that resilience is associated with lower anxiety levels. This finding agreed with previous research conducted during the COVID-19 pandemic, which demonstrated a significant correlation between resilience and anxiety experienced by healthcare workers [31]. Resilience has also shown a protective effect on anxiety and mental health [32], with more resilience indicating better mental health. Resilience reflects an individual’s ability to ‘bounce back’ in difficult circumstances [15], and it is vital to the ability to cope with a crisis, such as COVID-19. As resilience can be developed [e33], it is necessary to develop interventions to promote resilience among nurses to prevent or mitigate the occurrence of psychological distress during pandemic situations. Nevertheless, our finding revealed that attendance at resilience workshops was not significantly associated with anxiety or depression, although resilience itself was. This finding may imply that attendance at a resilience workshop alone was inadequate to enhance resilience. Indeed, a previous meta-analytic review [33] demonstrated that programs employing a one-on-one delivery format were most effective, followed by the classroom-based group delivery format and train-the-trainer and computer-based delivery formats. Thus, ways of building resilience should be further explored and expanded to improve effectiveness.

Emerging evidence has shown that mindfulness is a contributing factor to resilience [34,35]. Thus, interventions incorporating elements of mindfulness could increase resilience [36,37], mitigate fatigue and burnout [38], and cultivate psychological well-being [39,40]. In the current survey, nearly 40% of the respondents showed interest in the mindfulness-training workshops. It has been suggesting that nursing programs incorporating resilience should be a regular part of training and continuing education for frontline healthcare workers [41,42]. Future studies and courses at the institutional level could adopt mindfulness training to build and sustain resilience, thereby reducing psychological distress among frontline healthcare workers.

Exhaustion, a stress-related factor, significantly predicted the level of anxiety. This is supported by studies conducted in Asia that have shown a correlation of psychological distress with exhaustion [43,44]. These findings could be explained by the physical and psychological exhaustion that occur as a result of work commitments and fear of COVID-19 infection. These frontline nurses failed to have sufficiently full personal lives or effective methods to provide stress relief. It highlights the need for academic institutes and organizations to improve the stress levels and mental health of frontline nurses during the pandemic by acknowledging and relieving their stress.

Interestingly, anxiety about the infection, another stress-related factor, was significantly positively associated with the level of depression. This finding is consistent with those previous studies conducted among the general population in Hong Kong [3] and healthcare professionals in South Asian country [45], which demonstrated that people with depressive symptoms reported having a significant fear of COVID-19. This fear may be attributed to the severity and unpredictable trajectory of the disease, as well as information overload or misinformation about the pandemic [45]. In addition, the fear of COVID-19 might possibly be worsened by the co-existence of depression and anxiety disorder [46].

This study has several limitations. First, the respondents were full-time nurses who studied part-time at a single institution, which affects the generalizability of the findings. Second, reverse causality and reporting bias are possible due to the cross-sectional nature and self-reported measurement. Third, the response rate of 32% reported in this study was relatively low; although it was comparable to other online surveys, our survey may have been subject to response bias. Moreover, respondents who chose to participate may not be representative of non-participating individuals; for example, the non-participating individuals may not have direct experience of COVID-19 or online learning as major stressors, or they may have been too stressed to respond. In addition, other factors that were not related to work should be explored if they may account for the self-report measures, and levels of anxiety and depression, such as personality traits, e.g., neuroticism, overestimation of threat; family issues, e.g., family members being unemployed, caring children and elderly at home, parents being hospitalized, loss of loved ones; frequency of exposure to social media, etc. Only with a complete account of the factors affecting levels of psychological distress should a tailor-made intervention be considered, in which resilience training and mindfulness may not be the most appropriate approach to be adopted. Although the adjusted models demonstrated that part-time study was not significantly associated with the psychological distress of this study cohort, the lack of a comparison group who was not having part-time study might limit the generalization of the findings. In the future study, a comparison group should be considered in order to assess if part-time study was a factor affecting levels of psychological distress of the study population.

## 5. Conclusions

To conclude, licensed full-time practicing nurses undertaking part-time studies in higher education appeared to have a mild level of anxiety on average. The resilience score and exhaustion were significantly associated with anxiety, and anxiety about infection was significantly associated with depression. Effective measures and interventions should be developed at the organizational level and within the academic sector to alleviate the psychological distress experienced by licensed full-time practicing nurses studying part-time in higher education in facing the pandemic.

## Figures and Tables

**Table 1:** Sociodemographic and other characteristics of the subjects (*n* = 124).

	*n*	%
Program of study		
Bachelor’s degree	64	51.6
Master’s degree	60	48.4
Year of study		
Year 1	49	39.5
Year 2	75	60.5
Age group (years)		
20–25	26	21.0
26–30	59	47.5
31–35	26	21.0
≥36	13	10.5
Sex		
Male	16	12.9
Female	108	87.1
Marital status		
Single	97	78.2
Married	25	20.2
Divorced/widowed	2	1.6
Post-registration working experience (years)		
<1	11	8.9
1–5	62	50.0
6–10	35	28.2
11–15	7	5.6
>15	9	7.3
Work place		
Public hospital	92	74.2
Private hospital	9	7.3
Department of Health	19	15.3
Others	4	3.2
Number of household members lived with		
0	3	2.4
1	15	12.1
2	29	23.4
3	42	33.9
4	24	19.4
≥5	11	8.9
Hours spent on online learning per week		
0–8	66	53.2
9–16	46	37.1
17–25	5	4.0
≥26	7	5.6
Average number of days in contact with suspected or confirmed COVID-19 cases		
<14	67	54
14–30	17	13.7
31–60	14	11.3
≥60	26	21
Previously attended mindfulness or resilience building workshop		
Yes	17	13.7
No	107	86.3
Interested in attending mindfulness or resilience building workshop		
Yes	48	38.7
No	76	61.3

**Table 2:** Descriptive statistics of the mean GAD-7, PHQ-2, resilience, stress-related factors, avoidance coping, and approach coping scores (*n* = 124).

	Mean (SD)	Minimum	Maximum
GAD-7 (possible range: 0–21)	6.14 (4.02)	0	15
PHQ-2 (possible range: 0–6)	1.80 (1.28)	0	5
Resilience (possible range: 0–40)	23.53 (5.59)	6	40
Stress-related factors:			
Anxiety about infection (possible range: 0–27)	12.27 (5.06)	0	25
Exhaustion (possible range: 0–15)	5.92 (2.95)	0	12
Workload (possible range: 0–6)	3.59 (1.57)	0	6
Feeling protected (possible range: 0–9)	2.64 (1.56)	0	7
Part-time study (possible range: 0–6)	3.03 (1.16)	0	6
Coping:			
Avoidance (possible range: 12–48)	23.33 (4.95)	12	36
Approach (possible range: 12–48)	31.14 (5.39)	14	43

SD: Standard deviation, GAD: Generalized anxiety disorder, PHQ: Patient Health Questionnaire.

**Table 3:** Linear regression of GAD-7 on stress-related factors, resilience, coping, and sociodemographic factors.

--	Unadjusted Model		Adjusted Model	
	Coefficient (95% Confidence Interval)	*p* Value	Coefficient (95% Confidence Interval)	*p* Value
Program of study				
Bachelor’s degree	0.879 (−0.548, 2.307)	0.225	−0.476 (−1.839, 0.888)	0.490
Master’s degree (reference)	0		0	
Year of study				
Year 1 (reference)	0		0	
Year 2	−0.246 (−1.713, 1.222)	0.741	0.044 (−1.166, 1.255)	0.942
Age group (years)				
22–25	2.192 (−0.496, 4.880)	0.218	−3.420 (−7.826, 0.986)	0.300
26–30	1.833 (−0.591, 4.258)	0.109	−3.323 (−7.452, 0.806)	0.127
31–35	0.538 (−2.149, 3.226)	0.137	−3.993 (−8.149, 0.163)	0.113
≥36 (reference)	0	0.692	0	0.059
Sex				
Male	−0.157 (−2.298, 1.983)	0.885	−0.791 (−2.624, 1.042)	0.393
Female (reference)	0		0	
Marital status		0.164		0.517
Single (reference)	0		0	
Married	−1.575 (−3.348, 0.199)	0.081	−0.506 (−2.435, 1.422)	0.603
Divorced/widowed	−2.495 (−8.143, 3.154)	0.384	−3.271 (−9.026, 2.484)	0.262
Post-registration work experience (years)		0.063		0.466
<1 (reference)	0		0	
1–5	−1.799 (−4.351, 0.752)	0.165	−1.374 (−3.676, 0.928)	0.239
6–10	−2.792 (−5.488, −0.096)	0.042	−1.343 (−4.095, 1.410)	0.335
11–15	−4.792 (−8.563, −1.021)	0.013	−3.154 (−7.825, 1.517)	0.183
>15	−3.697 (−7.202, −0.192)	0.039	−5.103 (−10.815, 0.609)	0.079
Workplace		0.211		0.743
Public hospital (reference)	0		0	
Private hospital	0.321 (−2.442, 3.084)	0.818	1.346 (−1.055, 3.747)	0.268
Department of Health	−2.088 (−4.082, −0.095)	0.040	0.298 (−2.630, 3.225)	0.840
Others	−0.707 (−4.747, 3.334)	0.730	0.227 (−3.107, 3.561)	0.893
Number of household members lived with		0.477		0.591
0	−1.733 (−6.778, 3.312)	0.498	0.582 (−3.965, 5.129)	0.800
1 (reference)	0		0	
2	0.830 (−1.707, 3.367)	0.518	−0.617 (−2.833, 1.600)	0.582
3	1.910 (−0.490, 4.309)	0.118	0.266 (−1.736, 2.268)	0.792
4	1.100 (−1.526, 3.726)	0.408	−0.204 (−2.326, 1.918)	0.849
≥5	0.661 (−2.506, 3.827)	0.680	−1.797 (−4.549, 0.954)	0.198
Hours spent on online learning per week		0.857		0.068
0–8 (reference)	0		0	
9–16	−0.642 (−2.185, 0.902)	0.412	0.086 (−1.184, 1.355)	0.894
17–25	−0.824 (−4.551, 2.902)	0.662	−0.485 (−3.599, 2.628)	0.757
≥26	−0.281 (−3.475, 2.912)	0.862	3.937 (0.897, 6.976)	0.012
Average days in contact with suspected or confirmed COVID-19 cases		0.260		0.722
<14 (reference)	0		0	
14–30	1.976 (−0.176, 4.129)	0.072	0.992 (−0.760, 2.744)	0.263
31–30	0.174 (−2.156, 2.503)	0.883	−0.047 (−1.957, 1.864)	0.961
≥60	1.119 (−0.713, 2.950)	0.229	0.192 (−1.487, 1.871)	0.821
Ever attended mindfulness or resilience building workshop				
Yes	−1.318 (−3.391, 0.756)	0.211	−1.156 (−3.100, 0.787)	0.240
No (reference)	0		0	
Resilience score	−0.371 (−0.481, −0.260)	<0.001	−0.188 (−0.327, −0.050)	0.008
Avoidance coping score	0.269 (0.132, 0.406)	<0.001	0.121 (−0.029, 0.271)	0.111
Approach coping score	−0.029 (−0.162, 0.105)	0.672	−0.013 (−0.159, 0.133)	0.859
Stress-related factors (scores)				
Anxiety about infection	0.427 (0.307, 0.547)	<0.001	0.053 (−0.129, 0.236)	0.564
Exhaustion	0.875 (0.687, 1.062)	<0.001	0.612 (0.298, 0.925)	<0.001
Workload	1.225 (0.821, 1.629)	<0.001	0.077 (−0.454, 0.607)	0.775
Feeling of being protected	−0.358 (−0.816, 0.100)	0.125	−0.174 (−0.613, 0.265)	0.433
Part-time study	1.438 (0.873, 2.002)	<0.001	0.117 (−0.557, 0.790)	0.731

**Table 4:** Linear regression of PHQ-2 on stress-related factors, resilience, coping, and sociodemographic factors.

	Unadjusted Model		Adjusted Model	
	Coefficient (95% Confidence Interval)	*p* Value	Coefficient (95% Confidence Interval)	*p* Value
Program of study				
Bachelor’s degree	0.191 (−0.264, 0.645)		−0.206 (−0.731, 0.318)	
Master’s degree (reference)	0	0.408	0	0.436
Year of study				
Year 1 (reference)	0		0	
Year 2	−0.097 (−0.562, 0.368)	0.680	−0.027 (−0.493, 0.439)	0.910
Age group (years)		0.111		0.595
22–25	0.615 (−0.231, 1.462)	0.153	0	
26–30	0.598 (−0.165, 1.362)	0.123	−0.011 (−1.707, 1.684)	0.989
31–35	0.000 (−0.847, 0.847)	1.000	0.004 (−1.585, 1.592)	0.996
≥36 (reference)	0		−0.484 (−2.083, 1.115)	0.549
Sex				
Male	0.375 (−0.301, 1.051)		0.230 (−0.476, 0.935)	
Female (reference)	0	0.274	0	0.519
Marital status		0.142		0.681
Single (reference)	0		0	
Married	−0.558 (−1.119, 0.004)	0.052	−0.328 (−1.070, 0.414)	0.382
Divorced/widowed	−0.418 (−2.206, 1.371)	0.645	−0.276 (−2.491, 1.939)	0.805
Post-registration work experience (years)		0.098		0.860
<1 (reference)	0		0	
1–5	−0.658 (−1.471, 0.154)	0.111	−0.482 (−1.368, 0.404)	0.283
6–10	−0.917 (−1.776, −0.058)	0.037	−0.388 (−1.447, 0.671)	0.469
11–15	−1.545 (−2.747, −0.344)	0.012	−0.246 (−2.043, 1.551)	0.786
>15	−0.990 (−2.107, 0.127)	0.082	−0.399 (−2.597, 1.799)	0.719
Workplace		0.227		0.876
Public hospital (reference)	0		0	
Private hospital	−0.146 (−1.023, 0.731)	0.742	0.125 (−0.799, 1.049)	0.789
Department of Health	−0.608 (−1.241, 0.024)	0.059	0.246 (−0.881, 1.372)	0.666
Others	−0.674 (−1.956, 0.608)	0.300	−0.413 (−1.696, 0.870)	0.524
Number of household members lived with		0.882		0.817
0	−0.267 (−1.885, 1.352)	0.745	−0.283 (−2.033, 1.466)	0.748
1 (reference)	0		0	
2	0.193 (−0.621, 1.007)	0.639	−0.429 (−1.282, 0.423)	0.320
3	0.376 (−0.394, 1.146)	0.335	−0.129 (−0.900, 0.641)	0.739
4	0.150 (−0.692, 0.992)	0.725	−0.390 (−1.206, 0.427)	0.346
≥5	0.036 (−0.979, 1.052)	0.944	−0.571 (−1.630, 0.487)	0.286
Hours spent on online learning per week		0.458		0.851
0–8 (reference)	0		0	
9–16	−0.053 (−0.538, 0.433)	0.830	0.166 (−0.322, 0.655)	0.501
17–25	−0.479 (−1.652, 0.694)	0.421	−0.247 (−1.446, 0.951)	0.683
≥26	−0.736 (−1.741, 0.269)	0.150	0.231 (−0.938, 1.401)	0.695
Average days in contact with suspected or confirmed COVID-19 cases		0.598		0.904
<14 (reference)	0		0	
14–30	0.226 (−0.463, 0.914)	0.518	−0.090 (−0.764, 0.584)	0.792
31–30	0.343 (−0.402, 1.089)	0.364	0.172 (−0.563, 0.907)	0.643
≥60	0.343 (−0.243, 0.929)	0.248	0.157 (−0.489, 0.803)	0.630
Ever attended mindfulness or resilience building workshop				
Yes	−0.312 (−0.971, 0.348)		−0.079 (−0.827, 0.669)	
No (reference)	0	0.351	0	0.834
Resilience score	−0.099 (−0.135, −0.062)	<0.001	−0.032 (−0.085, 0.021)	0.237
Avoidance coping score	0.048 (0.003, 0.094)	0.036	0.008 (−0.049, 0.066)	0.771
Approach coping score	−0.032 (−0.074, 0.010)	0.130	−0.019 (−0.075, 0.037)	0.509
Stress-related factors (scores)				
Anxiety about infection	0.122 (0.083, 0.162)	<0.001	0.071 (0.001, 0.141)	0.048
Exhaustion	0.210 (0.142, 0.278)	<0.001	0.109 (−0.012, 0.229)	0.076
Workload	0.347 (0.215, 0.479)	<0.001	0.044 (−0.160, 0.249)	0.667
Feeling of being protected	−0.191 (−0.334, −0.048)	0.009	−0.151 (−0.320, 0.018)	0.079
Part-time study	0.342 (0.156, 0.529)	<0.001	−0.071 (−0.330, 0.188)	0.588

## Data Availability

The data presented in this study are available on request from the corresponding author. The data are not publicly available due to its huge size and participants’ privacy protection.
